# The Liquor Habit

**Published:** 1893-09

**Authors:** C. T. Pope

**Affiliations:** Louisville, Ky.


### THE LIQUOR HABIT.

BY C. T. POPE, M. D., LOUISVILLE, KY.

In these days, when the curing of drunkards has loomed up into such prominence, my experience may be to some extent interesting, and at the same time the means of causing the profession to become more familiar with a remedy which, in my hands, has proven itself of much value.

For some time after the question of treating the liquor habit had begun to be agitated, I read numerous articles on the subject in both the medical and the secular press, but gave but little consideration to the claims made for the different cures (as they are called) until a patient came under my observation who, I thought, would make a good subject to experiment upon.

At about the same time my attention was called to the treatment recommended by Dr. S. H. Garvin, of this city, with whom I was personally acquainted, and who I knew had had ample opportunity to study the matter thoroughly. After a consultation with the doctor, I concluded to follow his advice in the treatment of my patient. The directions were: "Antidipsole" (as he calls his remedy), one teaspoonful six times a day. The meals—three a day—to be at stated hours, so as not to interfere with the medicine. Three warm baths a week, to be followed by a thorough rubbing-down with a coarse linen towel, to invigorate the skin, and rest in bed at least eight hours out of the twenty-four, to give repose to the nervous system. The result will be seen by a perusal of the case referred to, and also two others that came to me during the following three months:

Case 1. H. W., age forty-five, married, occupation at present clerk for a lumber merchant; was formerly a dealer himself, but dissipation caused his downfall, and for several years he had made only a bare subsistence for his family. When called to see him last October he was suffering from his fourth attack of mania-a-potu. As soon as he recovered, and was in proper condition, I put him on "Antidipsole." His anxiety to give up drink prompted him to give careful attention to my directions, and in three days all desire for liquor had passed away—a condition he had not known since he became addicted to its use. He soon began to relish his food, and to sleep well, and by the end of the month (the time required for the treatment) he was completely restored, and able to attend to business as well as ever. He has taken no intoxicating liquor of any kind since, nor has he had any desire to.

Case 2. M. W., age thirty-eight, single, book-keeper by profession, came to me for medical advice last November. The object of his visit was to have me dress a broken nose, the result of a drunken broil. During the time he was coming to my office to have his nose attended I learned from him his history. According to his statement, he had been a constant and heavy drinker ever since he was twenty years old, and the rough usage he had received during his last spree had about determined him to let whiskey alone for the future. At my suggestion to be treated for his bad habit he readily consented, and I immediately put him on "Antidipsole." His experience was similar to that of Case 1. The craving for alcoholic stimulants left him in a few days; he regained his appetite for food, and after a week's treatment he informed me that he had, the night previous, slept eight hours without interruption--something he had not done before since he began the use of intoxicants. He is a sober man to-day, and making a good salary at his calling.

Case 3. I. L., age forty-three, married, by trade a tinner, a periodical drinker whose sprees were usually continued until terminated by an attack of mania-a-potu. In January last I was called to see him in one of these attacks, which was the winding up of a spree that had lasted for several weeks. On his recovery, by the request of friends, himself consenting, I took him in hand to cure him of the liquor habit. "Antidipsole" was given him in teaspoonful doses six times a day. His faithfulness in carrying out the treatment was remarkable, and the result was that he was soon so completely relieved from his thirst for stimulants that he had no farther trouble from that source. Like the preceding two patients, restoration to the normal condition was rapid, and after the termination of the treatment he was, as he expressed himself, in good health, and no longer a slave to "King Alcohol."

It is not my purpose in this paper to enter into a discussion on the pathology of alcoholism, nor to express an opinion as to whether inebriety is a disease or only a habit, but simply to add my experience, with a few brief comments to this much discussed question.

Referring to the cases cited, what has been accomplished by the treatment for these patients? First, the craving for stimulants has been overcome, and thus, by enabling the patients to discontinue the imbibition of alcohol, the emunctories have been given an opportunity to throw off the poison existing in the system, and allow the various organs to perform their functions normally. The brain consequently being relieved, refreshing sleep was had, which rest resulted in more activity of that organ, and necessarily brightened intellect. So with the digestive apparatus: food which before was only taken irregularly and in small quantities, was now called for and relished, and in turn gave strength and tone to the general system, so that the exertion necessitated by the requirements of a busy life, which, while the individual was under the influence of alcohol, was performed with difficulty, was now accomplished with comparative ease.

But this improvement in the health and condition of the patient, while largely due to, was not altogether the result of giving up the use of intoxicants and the elimination of alcohol from the system. The remedy did more than simply relieve the craving for stimulants, as can be readily seen from its composition as shown by the formula: Lupulin, serpentaria, apium graviolens, cinchona, capsicum, and aromatics. The apium graviolens and lupulin are most excellent nervous stimulants, the capsicum the best of stimulating tonics for the digestive organs; and the cinchona and serpentaria rank, as diversified tonics, among the first. Here we have just what is called for in the general debility caused by inebriety, and the rapid recovery from the exhausted state which marks this condition, the restoration to a healthy appetite, refreshing sleep, and a general feeling of comfort is undoubtedly due, in a great measure, to the direct action of the medicine, and not altogether to the discontinuance of the stimulant.

What constitutes a cure of the liquor habit is a pertinent question, and one which is difficult to answer. Let us go back and look over our cases, and see what has been accomplised. Case 1, H. W. drank to such an extent for several years that he had made but a bare subsistence for his family. Commenced the treatment October, 1892; has now been sober eight months. Case 2, M. W. had been a regular drinker for eighteen years. Commenced treatment last November—now seven months since he took a drink. Case 3, I. L., a periodical drinker who was seldom sober, has not drunk liquor since last January—five months.

These individuals have not only not used stimulants since their treatment, but they have no inclination to do so. Are they cured? I answer, most certainly they are; the treatment has restored them to their normal condition, which is all that can be accomplished by medicine, and it is with themselves only whether they will continue to abstain from alcoholic beverages.

All talk about cures that will cause one who has taken them to loathe liquor is the most perfect bosh, and is only given out to catch the gullible. If after treatment the individual has lost all craving for stimulants, and in point of health has been perfectly restored, has not enough pride to hold himself above temptation, he will drift back into his old habit, no matter who treats him, or how he is treated.

And now, in conclusion, let me say a few words in regard to the remedy, "Antidipsole." The combination of medicines for the condition for which it was formulated is certainly a very happy one, for while there is no doubt a large percentage of those who take it for the liquor habit will be cured, all will be benefitted, and none can possibly be harmed, for nothing of a dangerous character enters into its composition—nothing that could possibly do injury.—*From American Practitioner and News, July 1st*, 1893.

